# Supplementary material for: Comparison of the Cox Proportional Hazards Model and Random Survival Forest Algorithm for Predicting Patient‐Specific Survival Probabilities in Clinical Trial Data
Source: Biom J. 2026 Sep 25;68(5):e70171. doi: 10.1002/bimj.70171 (PMC13615326; doi:10.1002/bimj.70171)
Supplement: Supplementary file 2 [file BIMJ-68-e70171-s002.zip › 2_local_analysis/Byar/figures/cens_03/FigC6a_Byar_03_calibration_main_100_beta_04_scale_shape_1.pdf]

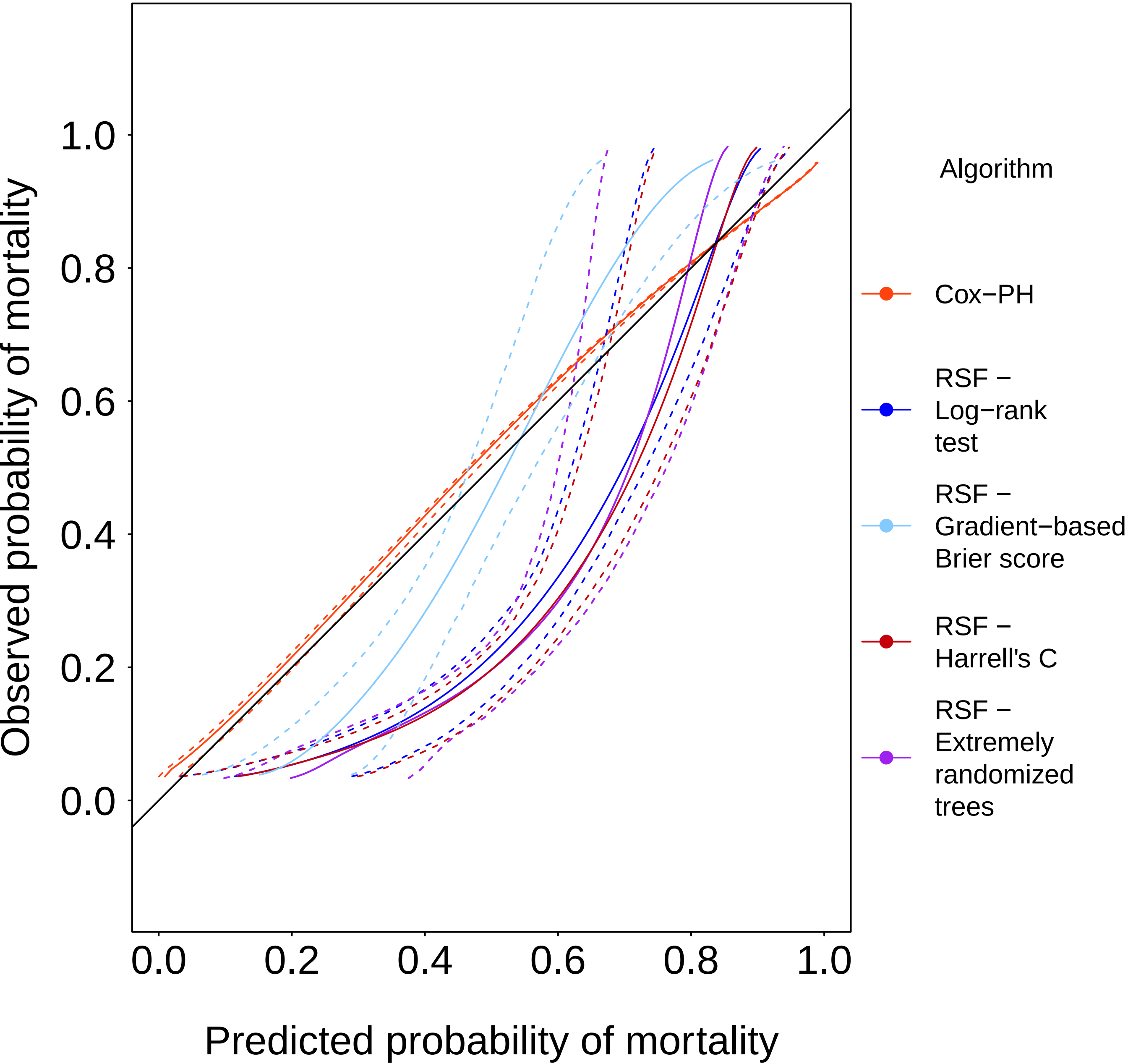

Observed probability of mortality
1.0
0.8
0.6
0.4
0.2
0.0
0.0
0.2
0.4
0.6
0.8
1.0
Predicted probability of mortality
Algorithm
Cox−PH
RSF − Log−rank test
RSF − Gradient−based Brier score
RSF − Harrell's C
RSF − Extremely randomized trees
